# Hybrid-Type SELEX for the Selection of Artificial Nucleic Acid Aptamers Exhibiting Cell Internalization Activity

**DOI:** 10.3390/pharmaceutics13060888

**Published:** 2021-06-15

**Authors:** Hiro Uemachi, Yuuya Kasahara, Keisuke Tanaka, Takumi Okuda, Yoshihiro Yoneda, Satoshi Obika

**Affiliations:** 1 National Institutes of Biomedical Innovation, Health and Nutrition (NIBIOHN), Osaka 567-0085, Japan; hao-ding@ds-pharma.co.jp (H.U.); tanaka-kei@phs.osaka-u.ac.jp (K.T.); taku0903taku@gmail.com (T.O.); y-yoneda@nibiohn.go.jp (Y.Y.); obika@phs.osaka-u.ac.jp (S.O.); 2 Graduate School of Pharmaceutical Sciences, Osaka University, Osaka 565-0871, Japan; 3 Oncology Laboratory for Discovery Chemistry & Technology, Sumitomo Dainippon Pharma Co., Ltd., Osaka 554-0022, Japan

**Keywords:** artificial nucleic acid aptamer, hybrid-SELEX, internalization, TROP2, drug delivery, oligonucleotide, base-modified artificial nucleic acid, capillary electrophoresis

## Abstract

Nucleic acid aptamers have attracted considerable attention as next-generation pharmaceutical agents and delivery vehicles for small molecule drugs and therapeutic oligonucleotides. Chemical modification is an effective approach for improving the functionality of aptamers. However, the process of selecting appropriately modified aptamers is laborious because of many possible modification patterns. Here, we describe a hybrid-type systematic evolution of ligands by exponential enrichment (SELEX) approach for the generation of the artificial nucleic acid aptamers effective against human TROP2, a cell surface protein identified by drug discovery as a promising target for cancer therapy. Capillary electrophoresis SELEX was used for the pre-screening of multiple modified nucleic acid libraries and enrichment of TROP2 binding aptamers in the first step, followed by functional screening using cell-SELEX in the second step for the generation of cell-internalizing aptamers. One representative aptamer, Tac-B1, had a nanomolar-level affinity to human TROP2 and exhibited elevated capacity for internalization by cells. Because of the growing interest in the application of aptamers for drug delivery, our hybrid selection approach has great potential for the generation of functional artificial nucleic acid aptamers with ideal modification patterns in vitro.

## 1. Introduction

Nucleic acid aptamers are single-stranded oligonucleotides that are functionally comparable to antibodies. Aptamers can be obtained through the in vitro screening of random nucleic acid libraries by the process known as the systematic evolution of ligands by exponential enrichment (SELEX) [1,2]. The SELEX selection process mainly includes three steps: (1) separation of sequences bound to the target from a mixture of random oligonucleotide libraries and the target molecule, (2) PCR amplification of target-bound sequences, and (3) purification of single-stranded oligonucleotides for the next round of selection. The separation step is critical for the identification of the desired aptamer and has the predominant influence on the selection time and cost. A variety of separation methods have been developed, including conventional protein bead-based SELEX [3], capillary electrophoresis (CE) SELEX [4,5,6,7], microfluidic SELEX [8,9], and flow cytometry SELEX [10]. In particular, CE-SELEX allows generation of aptamers in a short time because it has an extremely high partitioning efficiency despite the lack of pre-immobilization of the target protein. The protein bead-based SELEX enables isolation of a variety of aptamers with high affinity [11]; however, it has been proved that aptamers selected based on the levels of purified proteins alone may not recognize their target proteins in the physiological environment [12]. The 3D conformations and glycosylation levels of purified proteins can be different from those in the cell membrane [13]. To address this issue, cell-based SELEX was developed to generate aptamers against membrane proteins in their native form [14,15]. Furthermore, aptamers that bind to cell surface proteins can be rapidly internalized into cells, allowing researchers to conjugate secondary reagents, such as small molecule drugs [16,17], antisense oligonucleotides (ASOs) [18], or small interfering RNAs (siRNAs) [19] to develop aptamer-drug conjugates [20] for targeted drug delivery. Although some reports have described how cell-internalizing aptamers could be isolated using conventional SELEX, those methods did not guarantee the selection of functional aptamers [21]. To this end, Giangrande et al. introduced the cell internalization SELEX protocol that allowed them to eliminate aptamers lacking capacity for internalization and to obtain the desired aptamers from the inside of cells [22]. Furthermore, several aptamers have been generated using the hybrid-SELEX method [19,23,24]. A DNA aptamer specific for CD30 was generated using the hybrid-SELEX approach, which combines the cell-SELEX targeting the CD30-expressing lymphoma cells, and a subsequent selection with purified CD30 protein through the protein bead-based SELEX [19,23,24]. The hybrid-SELEX techniques enable identifying functional aptamers that could recognize their target of interest in the physiological environment.

In comparison to protein-based therapeutics, aptamers can be easily chemically modified for increased stability to nuclease degradation, enhanced target binding affinity, and improved pharmacokinetic properties [25,26]. The chemically modified aptamers are known as artificial nucleic acid aptamers. Due to their relatively low molecular weight, aptamers are considered to penetrate tissues more efficiently than antibodies [27]. Notably, the base modification strategy has significantly improved aptamer binding affinity and the success rate of SELEX [28]. In this approach, base-modified artificial nucleic acids are introduced by replacing the standard dTTP or dCTP with modified dUTP or dCTP, whose C5 position had additional functional groups, such as benzyl, imidazole, and indole, to mimic the side chain of natural amino acids. Our group also adopted this approach to discover artificial nucleic acid aptamers against a variety of targets [29,30,31]. This approach is powerful because it enables generating a large number of aptamers with increased affinity. However, the number of artificial nucleic acid libraries used in each SELEX process was limited, and their varieties were narrow, owing to the time-consuming iterative nature of the SELEX procedure. As SELEX commonly involves several rounds of selection, it is not realistic to test several different libraries. Moreover, when multiple bases are modified, the library varieties become more diverse [32]. Therefore, a simple and equitable method for pre-screening artificial nucleic acid libraries is required.

Human trophoblast antigen 2 (TROP2), also known as tumor-associated calcium signal transducer 2 (TACSTD2), is a type 1 cell surface protein belonging to the epithelial cell adhesion molecule family. TROP2 overexpression in various cancer cell lines has been reported to be associated with poor patient prognosis [33]. The anti-TROP2 antibody RS7 can be rapidly internalized into TROP2-expressing cells [34]. The antibody-drug conjugate sacituzumab govitecan targeting TROP2 was approved by the US Food and Drug Administration in 2020 as a third-line treatment for metastatic triple-negative breast cancer [35]. Aptamers are expected to have effects similar to those of antibodies, so their pharmacological actions are also of interest. However, to the best of our knowledge, cell internalization properties of anti-TROP2 aptamers have not yet been investigated.

In this study, we aimed to develop a new approach, a hybrid-type SELEX protocol, for the discovery of artificial nucleic acid aptamers with modification patterns that maximize the affinity to the target protein and allow efficient cell internalization. We selected TROP2 FcHis as the target protein for the development of hybrid-type SELEX protocols, as TROP2 is known to be internalized into the cell when an antibody binds to it, but it is unknown whether the binding of aptamers can trigger the internalization process. Hybrid-type SELEX comprises two selection methodologies: CE-SELEX and cell-SELEX. CE-SELEX was applied to pre-screen different libraries against the recombinant TROP2 FcHis protein and select TROP2-binding aptamers, owing to its high detection sensitivity and partitioning efficiency. After CE-SELEX, we carried out cell-SELEX for the selection of cell-internalizing aptamers exhibiting TROP2-binding affinity against TROP2-expressing MCF-7 cells. Our approach facilitates the discovery of functional aptamers for targeted drug delivery and has the potential to accelerate the research on artificial nucleic acid aptamers.

## 2. Materials and Methods

### 2.1. Chemical Reagents and Cell Lines

Native ssDNA library, DNA primers, and templates were purchased from Japan Bio Service Co., Ltd. (Saitama, Japan) or Invitrogen Co., Ltd. (Carlsbad, CA, USA). Human TROP2 fused to IgG1 FcHis was purchased from R&D Systems, Inc. (Minneapolis, MN, USA). Human IgG1 FcHis was purchased from Acro Biosystems (Newark, DE, USA). We purchased 2′-deoxy-5-(3-(3-(1H-indol-3-yl)propanamido)prop-1-en-1-yl)uridine-5′-triphosphate (also known as 5-[(3-indolyl)propionamide-*N*-allyl]-2′-deoxyuridine-5′-triphosphate, dU^trp^TP) and 5-(3-aminoprop-1-en-1-yl)-2′-deoxycytidine-5′-triphosphate (also known as 5-aminoallyl-2′-deoxycytidine-5′-triphosphate, dC^aa^TP) from TriLink Biotechnologies, Inc. (San Diego, CA, USA). The sequences used in this study are summarized in Table S1. The MCF-7 human breast cancer cells were generously provided by the Japanese Collection of Research Bioresources Cell Bank (JCRB, Osaka, Japan). Cells were cultured in the Dulbecco’s modified Eagle’s medium (DMEM, NacalaiTesque, Kyoto, Japan), supplemented with 10% fetal bovine serum (Biosera, Kansas City, MO, USA) and 100 U/mL penicillin and streptomycin (NacalaiTesque, Kyoto, Japan), and maintained at 37 °C in a humid atmosphere with 5% CO_2_.

### 2.2. Preparation of the Initial Artificial Nucleic Acid Aptamer Libraries

Library A (unmodified DNA library) was used without further purification. Library B was prepared by using single-primer PCR. The reaction mixture included template T2H (80 nM), 6-fluorescein amidite (FAM)-labeled forward primer P1F (20-mer, 0.4 μM), dATP (0.2 mM), dU^trp^TP (0.2 mM), dGTP (0.2 mM), dCTP (0.2 mM), KOD Dash DNA polymerase (0.005 U/μL, Toyobo Co. Ltd., Osaka, Japan), and a 1× polymerase reaction buffer. PCR reaction conditions were set at 94 °C for 30 s, 54 °C for 30 s, and 74 °C for 60 s. Twelve cycles were carried out using a thermal cycler (FUJIFILM Wako Pure Chemical Corporation, Osaka, Japan). Single-stranded modified DNA strands were separated via denaturing polyacrylamide gel electrophoresis (PAGE), using the phenomenon of band shift caused by the introduction of artificial nucleic acids. The resulting single-stranded modified DNA strands were used as the initial library for selection. For library C, the T2H template (1 μM) and P1F forward primer (0.8 μM) were mixed in 1×polymerase reaction buffer with dATP (0.2 mM), dU^trp^TP (0.2 mM), dGTP (0.2 mM), and dC^aa^TP (0.2 mM), heated at 95 °C for 1 min, and then allowed to cool slowly to 25 °C for 60 min. KOD Dash DNA polymerase (0.025 U/μL) was added to the mixture and allowed to start the extension reaction at 74 °C for 60 min. The resulting mixture was purified in the same manner as described above, to obtain library C.

### 2.3. Pre-Screening of Aptamer Libraries

All CE experiments were performed using a PA 800 Plus apparatus (AB Sciex Pte. Ltd., Framingham, MA, USA). The excitation and emission wavelengths were 488 and 520 nm, respectively. An eCAP fused-silica capillary [80.2 cm (70 cm effective length) × 70 µm internal diameter × 375 µm outside diameter, (Beckman Coulter, Brea, CA, USA)] was used. All solutions used for CE were filtered through a 0.22 µm filter. For preconditioning, the capillary was flushed with 0.1 M aqueous NaOH for 10 min, followed by sequential washing with deionized water and running buffer (100 mM sodium borate (pH 8.35) for 10 min, respectively. The native and modified DNA libraries were dissolved in the binding buffer to 1 μM (buffer composition: 20 mM Tris-HCl buffer [pH 7.4], 10 mM NaCl, 1 mM MgCl_2_, and 0.05% Tween 20), denatured by heating up to 95 °C for 1 min, and gradually cooled to 25 °C (0.47 °C/min). The human TROP2 FcHis protein (final concentration: 500 nM) was incubated with the library (final concentration: 500 nM) in the binding buffer at 37 °C for 30 min. The resulting mixture was stored at 15 °C before it was loaded onto a CE instrument. Samples were injected into the capillary using a pressure injection (0.5 psi, over 3.8 s), and a voltage of +18 kV was applied across the capillary for 25 min. The analysis was carried out using 32 Karat software (Beckman Coulter, Inc., Brea, CA, USA).

### 2.4. CE-SELEX Process

Incubation and separation experiments were conducted in the same manner as described above. For the first round of selection, the U^trp^-modified library (500 nM, sequence diversity: ~10^10^) was incubated with the recombinant human TROP2 FcHis protein (500 nM) for 30 min, followed by affinity separation using CE. For CE separation, the bound species were collected into a collection vial containing 50 µL of the running buffer. The collected CE samples were amplified by using PCR. The mixture contained forward primer P1P (0.3 µM, phosphate modification at the 5′ end), reverse primer P2H_C3 (0.4 µM, hexachloro-fluorescein modification at the 5′ end), dNTPs (0.2 mM each), KOD Dash DNA polymerase (0.005 U/µL), and 1× polymerase reaction buffer. Twenty PCR cycles under the conditions identical to those during library preparation were carried out. The amplicon was obtained by ethanol precipitation. The phosphate-modified sense strand was digested with λ-exonuclease (0.025 U/μL) in a 1× λ-exonuclease reaction buffer (New England Biolabs Japan Inc., Tokyo, Japan), at 37 °C for 60 min. The obtained single-stranded DNA (antisense strand) was desalted by ultrafiltration and used as template for the following single-primer PCR using dU^trp^TP in the same manner as described for library preparation. After purification via denaturing PAGE, a new U^trp^-modified library was used for the next round of selection.

### 2.5. Cell-SELEX Process

The selection scheme used in this study is summarized in Section 3.4. We named the first round of cell-SELEX as round 6b, because cell-SELEX started after the five rounds of CE-SELEX.

On day 1, MCF-7 cells were seeded onto a 6 cm dish (1.2 × 10^6^ cells/dish) and incubated at 37 °C in a humid atmosphere with 5% CO_2_. On day 2, the U^trp^-modified library (0.1 nmol) from round 5 of CE-SELEX was dissolved in the annealing buffer (1 mM MgCl_2_ in phosphate-buffered saline [PBS]). The resulting mixture was heated at 95 °C for 1 min, then allowed to cool slowly to 25 °C for 60 min to refold the aptamers. MCF-7 cells were washed with DMEM supplemented with 0.1 mg/mL yeast tRNA (Sigma Aldrich, St. Louis, MO, USA) [36], followed by the pre-incubation in the same medium for 5 min at 25 °C. The folded library solution was mixed in DMEM described above and incubated with MCF-7 cells at 37 °C for 60 min. After incubation, the cells were washed with PBS three times. The cells were then treated with DNase I (0.1 μg/μL, Sigma Aldrich) in the Hank’s balanced salt solution supplemented with 5 mM MgCl_2_ at 37 °C for 30 min. After an additional wash with PBS, treatment with DNAzol^®^ Direct reagent (Molecular Research Center, Inc., Cincinnati, OH, USA) was performed to recover the internalized aptamers according to the supplier’s instructions. The recovered aptamers were amplified by using PCR. A new modified library was synthesized by using the amplicon as template. The synthesis procedures were the same as described for library preparation. After purification of the library, the subsequent selection processes were continued until round 9b. To increase the stringency of the selection process in the later rounds of selection, the washing and incubation periods were modified (Table S2).

### 2.6. Sequencing and Bioinformatic Analysis of Aptamer Libraries

Libraries of aptamers obtained from rounds 1 to 9 and round 9b were sequenced using next generation sequencing (NGS) technology by using Miseq^TM^ (Illumina, Inc., San Diego, CA, USA) according to the supplier’s instructions. We added some adaptor sequences to each library via consecutive PCR to generate a dsDNA library that contains all samples for sequencing. The resulting dsDNA library for NGS was purified using a QIAquick PCR Purification Kit (Qiagen, Hilden, Germany) according to the manufacturer’s instructions. We used AptaSUITE, a software collection developed by Hoinka et al., for processing all raw data, aptamer clustering, and result visualization [37].

### 2.7. Preparation of Aptamers

Each artificial nucleic acid aptamer was synthesized using a method similar to that used for the modified library preparation. The forward primer P1F labeled at the 5′ end with 6-FAM was used. Complementary sequences for individual aptamers were purchased from Invitrogen and used as templates for single-primer PCR. After the reaction, the single-stranded aptamers were separated via denaturing PAGE using the phenomenon of band shift caused by the introduction of artificial nucleic acids. The concentration of each aptamer was calculated from the absorbance at 260 nm using the sum of molar absorption coefficients based on the sequence containing artificial nucleic acids.

### 2.8. Binding Affinity Analysis with CE

Affinity assays were performed using PA 800 Plus and an eCAP fused-silica capillary with a short length [30.2 cm (20 cm effective length) × 70 µm internal diameter × 375 µm outside diameter, (Beckman Coulter)]. Before the analysis, the capillary was sequentially washed with 0.1 M aqueous NaOH and deionized water. The binding buffer and running buffer were identical to those used in CE-SELEX. Each aptamer was dissolved in the binding buffer, denatured by heating to 95 °C for 1 min, and gradually cooled (0.47 °C/min) to 25 °C. The folded aptamer (10 nM) was incubated with TROP2 FcHis protein or FcHis tag protein at 37 °C for 30 min. The resulting mixture was stored at 15 °C until it was loaded onto a CE instrument. Samples were injected into the capillary using a pressure injection (0.5 psi, over 3.8 s), and a voltage of +12 kV was applied across the capillary for 4.5 min. Electropherograms were recorded and analyzed using 32 Karat software. The apparent dissociation constants (*K*_d_) were determined as described previously [6].

### 2.9. Cell Internalization Assays Using the qPCR Method

We seeded MCF-7 cells into 96-well plates at a density of 1.5 × 10^4^ cells/well. After 24 h, the cells were washed and pre-incubated with DMEM supplemented with 0.1 mg/mL yeast tRNA for 5 min. The individual aptamers or libraries (final concentration: 10 nM) were dissolved in the annealing buffer at 10-fold of the final concentration, folded as described above, and then mixed in DMEM containing 0.1 mg/mL yeast tRNA, followed by the incubation with MCF-7 cells at 37 °C for 60 min. After incubation, the solution was removed and the cells were washed with PBS three times. The cells were then treated with DNase I (0.1 μg/μL) in the Hank’s balanced salt solution supplemented with 5 mM MgCl_2_ at 37 °C for 30 min. The internalized aptamers were recovered by an additional wash with PBS followed by DNAzol^®^ Direct reagent treatment. Quantification of internalized aptamers was conducted by using qPCR (Applied Biosystems, Waltham, MA, USA) according to the supplier’s instructions. The standard curve method was used for the quantification of each aptamer based on the known concentrations of authentic aptamer samples. We prepared a serial dilution (five points, 10-fold dilution) of each authentic aptamer and used each serial dilution in qPCR measurements. The standard curve was generated by fitting Cq values against the log concentrations of authentic aptamers. The data were normalized to those of the initial library as control. All experiments were performed in biological triplicate.

### 2.10. Statistical Analysis

The quantitative data are expressed as the mean ± SD. Experiments were performed in triplicate. Data were compared using the two-sided Student’s *t*-test. Differences were considered to be statistically significant when the *p*-values were less than 0.05.

## 3. Results

### 3.1. Pre-Screening of Artificial Nucleic Acid Libraries Using CE

**Figure 1:**
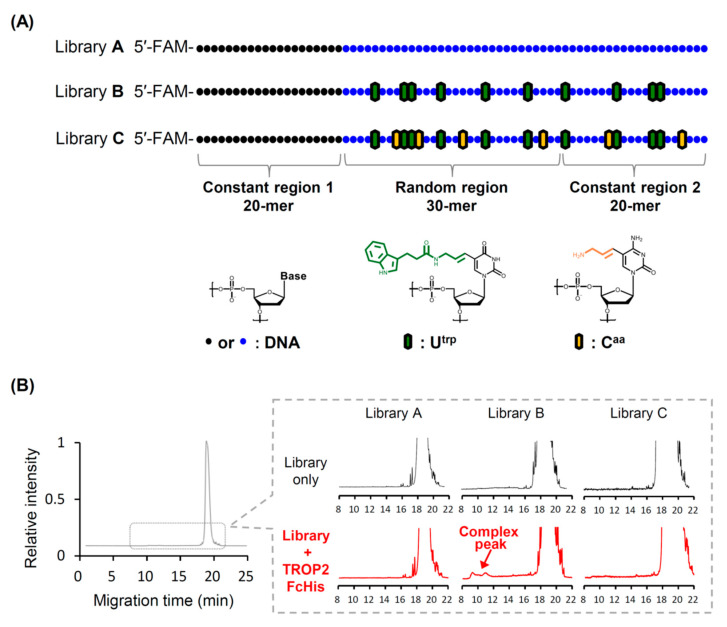
Pre-screening of artificial nucleic acid libraries using CE. (**A**) Schematic representation of three random DNA libraries with different modifications. Each library contained a 30-mer random region flanked by two constant regions at the 5′ and 3′ ends. (**B**) Capillary electropherograms for each library with or without human TROP2 FcHis protein are shown in the pre-screening experiment. All electropherograms recorded the fluorescent intensity of FAM-labeled aptamers versus migration time (min), and extremely small, complex peaks were detected by zooming into the relevant peak areas.

The following three types of DNA libraries were prepared for pre-screening: (A) native DNA library, (B) U^trp^-modified library made using dU^trp^TP instead of dTTP, and (C) U^trp^/C^aa^-modified library made using dU^trp^TP and dC^aa^TP instead of dTTP and dCTP, for library construction (Figure 1A).

All libraries had a 30-mer random region flanked by two 20-mer constant regions that acted as primer binding sites for PCR amplification. Library synthesis was accomplished using single-primer PCR or the primer-extension method. A 20-mer DNA primer that could hybridize to chemically synthesized DNA templates was elongated by KOD Dash DNA polymerase to generate modified libraries B and C. Owing to this synthesis method, the modified nucleotides were introduced randomly at positions 21–50 and 51–70 with the defined position of the entire sequence. To increase detection efficiency, all libraries were labeled with FAM at the 5′ end to ensure that the CE-based laser-induced fluorescence detection system could detect the aptamer-target binding event with high sensitivity. Each library was mixed with the recombinant TROP2 FcHis protein, and the resulting mixture was subjected to CE analysis. Although TROP2 can reportedly bind to a random DNA library with a relatively high level of affinity [38], only the U^trp^-modified library B showed a complex peak, indicating that it contains a higher amount of active species than other libraries (Figure 1B). Based on these pre-screening results, we chose to use the U^trp^-modified library B for the subsequent SELEX experiments.

### 3.2. CE-SELEX for Anti-TROP2 Aptamer Selection

**Figure 2:**
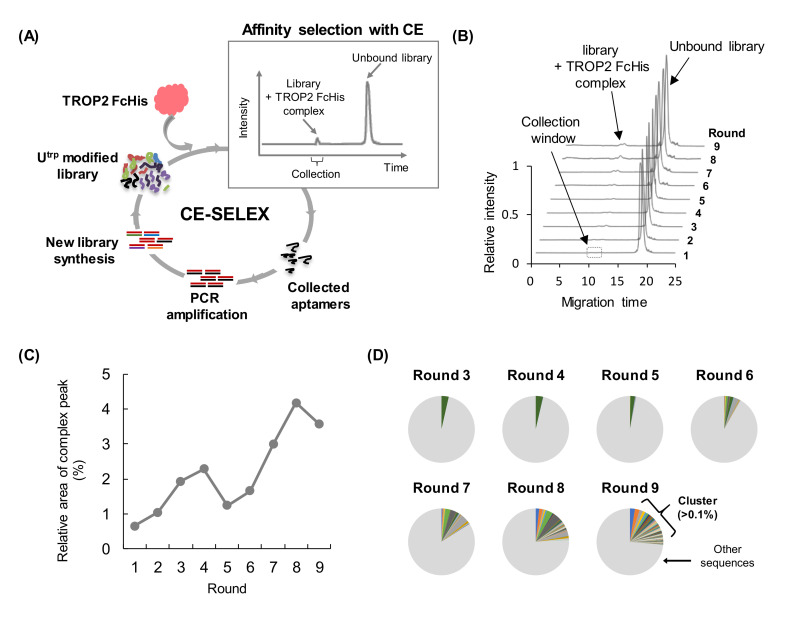
Selection of TROP2 binding aptamers using CE-SELEX. (**A**) CE-SELEX scheme for the selection of U^trp^ modified aptamers that can bind to the recombinant TROP2 FcHis protein. (**B**) Electropherograms were obtained from rounds 1 to 9 in the separation step. The target-binding species were collected during the collection window, indicated as a dotted line box, and amplified for the next round of selection. (**C**) Ratio of the complex peak to the unbound library peak in each round is shown. (**D**) Degree of sequence enrichment after two rounds of CE-SELEX. Clusters with more than 0.1% of the sequences in each round are visualized in colors, and all clusters with less than 0.1% of sequences are marked as other sequences (gray). Rounds 1 and 2 were omitted due to low clustering efficiency.

In our screening process, we planned to combine two distinct selection strategies, CE-SELEX and cell-SELEX, to obtain the desired aptamers. In the first strategy, we used CE-SELEX to enrich TROP2 binding aptamers (Figure 2A).

A TROP2 FcHis-fusion protein was used as the target protein in CE-SELEX, as described previously [38]. There is a possibility of obtaining non-specific FcHis binding aptamers [39]; and therefore, we evaluated the binding affinity of the aptamer candidates to both FcHis and TROP2 FcHis protein, for obtaining TROP2-specific aptamers. The progress of the selection process was monitored by measuring the ratio of the complex peak area to the unbound library in each round (Figure 2B). After two rounds of selection, the concentration of TROP2 FcHis was reduced to 200 nM to increase selection stringency. We confirmed the tendency of the complex peak area to increase, which suggested that the active species were enriched until round 9 of the selection process (Figure 2C). We observed a reduction of the complex peak in rounds 5 and 6. Based on the NGS data (Figure 2D), we concluded that rounds 5 and 6 were still in the intermediate stage of screening, that is to say, the sequence enrichment had not yet been completed. Therefore, small variations in manipulation could have a relatively large impact. For example, variability during CE fractionation or PCR bias may have possibly been involved [40]. High-throughput sequencing was performed using NGS, and the sequence data were organized, clustered, and visualized using AptaSuite, a user-friendly graphical user interface-based analytical tool for handling aptamer data [37,41]. We found that enrichment did not substantially increase in rounds 1–2 of selection (data not shown), but increased in the subsequent rounds (rounds 3–9) of CE-SELEX. After nine rounds of selection, the clusters with an abundance ratio of >0.1% were enriched over 26% of the total sequences, indicating that the use of CE-SELEX led to successful enrichment (Figure 2D).

### 3.3. Binding Affinity and Specificity of Aptamers Selected with CE-SELEX

*K*_d_ values of aptamer candidates that were highly enriched in round 9 were determined using a CE-based affinity assay. Given that the TROP2 FcHis protein used in this experiment had an additional FcHis tag, we used recombinant FcHis as a control protein. The most abundant sequence had a high affinity for TROP2; however, it also bound to FcHis (data not shown). Within the top 20 sequences, we observed that five sequences had a nanomolar-level affinity to TROP2 FcHis and high specificity towards the control protein (Table 1, Figure S1). We have confirmed that a random DNA library (library A) and a single-stranded DNA having unrelated sequence to the present study did not bind to both TROP2 FcHis and FcHis protein in this assay (Figure S2). We have also verified that the aptamer was TROP2-specific, using an electrophoretic mobility shift assay (Figure S3). In particular, Tac-A5 had the strongest binding affinity to TROP2 FcHis (*K*_d_ = 50 ± 6.9 nM) among the aptamer candidates. It also had 16 (53%) U^trp^ residues in the random region, which was the most abundant in U^trp^ residues among the selected sequences, perhaps because of the U^trp^ base-dependent gain in affinity in our case. Due to the relatively low abundance of Tac-A5 in the library, we thought that the exploration of sequences with a wider range could allow us to identify molecules that bind more strongly to TROP2 FcHis, but the number of aptamer candidates increased dramatically. Therefore, we decided to use the functional screening method cell-SELEX as a part of hybrid-type SELEX for effectively salvaging cell-internalizing aptamers.

### 3.4. Cell-SELEX for the Enrichment of Internalizing Aptamers against MCF-7 Cells

**Figure 3:**
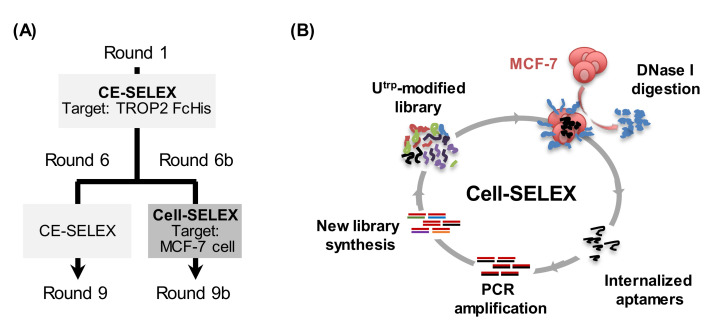
Details of hybrid-type SELEX and schematic representation of cell-SELEX. (**A**) The process flow of hybrid-type SELEX (the combination of CE-SELEX and cell-SELEX). Five rounds of CE-SELEX were performed against TROP2 FcHis protein, and then the selection process was classified into two types (CE-SELEX and cell-SELEX). CE-SELEX was performed from round 6 to 9, and cell-SELEX was performed from round 6b to 9b, to target MCF-7 cells. (**B**) The cell-SELEX scheme for the selection of U^trp^ modified aptamers that could be internalized into MCF-7 cells is shown. The cell surface-binding aptamers were digested using DNase I in the iterative selection flow.

Next, we added cell-SELEX to our selection process to enhance cell internalization of the aptamers (Figure 3A). The library from CE-SELEX round 5 was chosen as the initial library for cell-SELEX, because sequence enrichment had started from round 5, based on the CE and NGS analysis results (Figure 2C,D). Four additional rounds (rounds 6b to 9b) were performed to adopt the cell-SELEX protocol for use with MCF-7 cells, a human breast cancer cell line known to overexpress TROP2 [42] (Figure S4). The selection scheme used in this study is summarized in Figure 3B. Briefly, (1) in round 6b, the library from round 5 of CE-SELEX was incubated with MCF-7 cells in the culture medium, (2) cell surface-bound aptamers were digested by DNase I treatment, and (3) internalized aptamers were recovered and subjected to PCR amplification. After purification of the library, the subsequent selection processes were continued until round 9b. The number of washes increased and the period of incubation of the aptamer library with MCF-7 cells decreased gradually (Table S2). The library of the final round 9b (library 9b) was sequenced using NGS. The NGS data showed that these two different SELEXs had different selection biases (Figure S5). The aptamers Tac-A1–A5, which have a higher affinity, disappeared or their ranking dropped down in library 9b, suggesting that a certain functional selection bias existed in our cell-SELEX experiment. The shared sequences that exist in both library 9 from CE-SELEX and library 9b from cell-SELEX were extracted to evaluate their TROP2 binding affinity.

### 3.5. Binding and Specificity of Aptamers Selected with Cell-SELEX

*K*_d_ values of shared sequences in both CE-SELEX and cell-SELEX were evaluated using CE in the same manner as those from CE-SELEX. Finally, we found three sequences with nanomolar-level affinity to TROP2 FcHis and high specificity towards the FcHis protein. These new aptamers obtained from hybrid-type SELEX (Tac-B1 to Tac-B3) had a notable TROP2 FcHis binding affinity (*K*_d_ = 89–187 nM) with conserved specificity to the FcHis protein (Table 2, Figure S1). Interestingly, the increase in abundance in round 9b, compared to that in round 9, was observed in 2/3rd of the sequences. However, they had a relatively low U^trp^ ratio (~40%) in the random region, compared to the sequences obtained with CE-SELEX alone (Table 1).

### 3.6. Evaluation of Capacity to Undergo Cell Internalization

**Figure 4:**
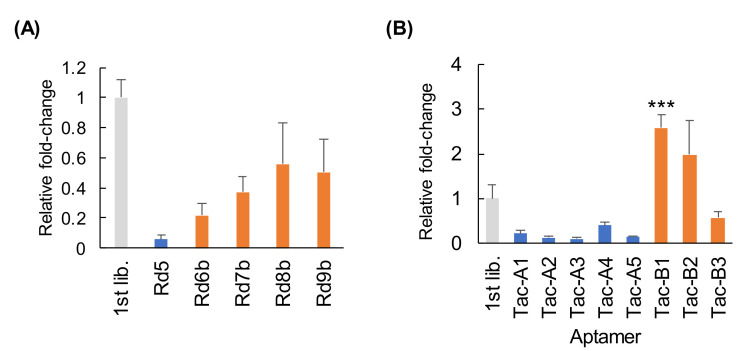
Evaluation of the degree of internalization of anti-TROP2 artificial nucleic acid aptamers by MCF-7 cells. (**A**) Evaluation of the internalization of each library obtained via CE-SELEX and cell-SELEX using MCF-7 cells. Each library was incubated with MCF-7 cells, washed, and treated with DNase I. Internalized species were quantified with qPCR. The degree of internalization was graphed as a ratio to that of the initial library (1st lib.). (**B**) Evaluation of the internalization of each aptamer obtained via CE-SELEX and hybrid-type SELEX. The evaluation was performed with qPCR using the same protocol as that used for libraries. These data represent the mean ± SD values of three independent experiments. Rd = Round. *** *p* < 0.001; two-sided Student’s *t*-test, compared to values for the 1st lib.

Finally, we evaluated the internalization potential of the aptamers isolated from CE-SELEX alone or using hybrid-type SELEX. The initial library (1st lib.) was used as negative control for the cell internalization assay. First, we evaluated cell internalization properties of each library obtained through hybrid-type SELEX. Library Rd5, obtained via five rounds of CE-SELEX alone, showed only approximately 1/10th of the cell internalization activity compared to that of the initial library, but as cell-SELEX was repeated, the cell internalization activity of each library steadily increased until round 9b (Figure 4A). Next, we evaluated cell internalization of each TROP2 aptamer in the same manner. As a result, the anti-TROP2 aptamers (Tac-A1–Tac-A5) obtained using CE-SELEX alone were found to have a low capacity for cell internalization despite their strong binding affinity, suggesting that their high binding activity may not guarantee their cellular activity. On the other hand, the aptamers selected using hybrid-type SELEX (Tac-B1–Tac-B3) showed a relatively high level of cell internalization (Figure 4B). Tac-B1 was internalized to the highest degree, with the level of internalization by cells being approximately 2.5-fold higher than that of the initial library used as control. Tac-B1, which was also observed in CE-SELEX alone, was ranked 56th in the enrichment efficiency ranking of library 9 derived via CE-SELEX. This was not a high position; therefore, it was estimated that it would take a lot of time and effort to find aptamers with such a functionality using CE-SELEX alone. These results demonstrate that the hybrid-type SELEX, which combines two distinct selection methodologies, could provide a valuable tool for the discovery of functional artificial nucleic acid aptamers in vitro.

## 4. Discussion

In this study, we have proposed a new approach for discovering artificial nucleic acid aptamers by utilizing the advantages of CE and conventional hybrid-SELEX technology. By using this approach, we have discovered new anti-TROP2 aptamers that could be internalized by cells. Our approach is suitable for obtaining artificial nucleic acid aptamers with both target protein-oriented modifications and optimal cellular internalization properties, and it may help accelerating drug delivery providing tailored artificial nucleic acid aptamers [43,44].

The artificial nucleic acid aptamers with increased functionality are in high demand in the field of drug discovery. Nucleobase modification is a useful approach for improving aptamer binding affinity. For example, amino acid side chain-like modifications were introduced at the 5-position of uridine; this resulted in a significant improvement in binding affinities and binding kinetics. This approach is known as slow off-rate modified aptamer technology and is one of the most successful achievements in the development of modified aptamers by SomaLogic [45]. In the present study, we also confirmed the effects of chemically modified aptamers on binding affinities when carrying out the pre-screening experiment. The U^trp^-modified library was found to have a notably enhanced binding activity over the natural DNA library (Figure 1B). The indole moiety at the C5 position of U^trp^ was postulated to mimic the side chain of tryptophan, which is considered to be a key residue in protein-protein interactions [46,47]. Furthermore, we found that the U^trp^-modified aptamers obtained from hybrid-type SELEX had relatively high propensity to be internalized by cells. Our recent reports also demonstrated the utility of the U^trp^ modification in the discovery of aptamers with high capacity for cell internalization for oligonucleotide and drug delivery in several cell types, including A549 and lung fibroblasts LL97A cells [48,49].

Currently, the selection of aptamers that could be internalized by cells is carried out predominantly by cell-SELEX and hybrid-SELEX [15,23,24]. The availability of cell-internalizing aptamers that target cancer cell antigens, such as HER2 [22], EGFRvIII [50], and nucleolin [51,52], has enabled the development of various aptamer-based therapeutic agents for targeted oligonucleotide delivery. Although cell-SELEX has great potential for aptamer selection, it has some drawbacks, such as difficulties in the optimization of experimental conditions and identification of targets. The main advantage of the conventional hybrid-SELEX is the ability to enrich for aptamers that display binding affinity for a specific target protein and have a specific property, such as cell internalization capacity, in the cell system. As mentioned earlier, there are great expectations for the development of artificial nucleic acid aptamers. Many of the conventional hybrid-SELEX methods start with cell-SELEX, which is labor-intensive; and when there are multiple libraries with different modification patterns, it becomes very time-consuming; therefore, making it difficult to identify the desired aptamers [21]. In order to overcome this limitation, we thought it was necessary to develop new hybrid-type SELEX protocols that enable extraction of a library with the highest probability of success for a target protein from several different artificial nucleic acid libraries in the early stage of selection, and subsequent identification of aptamers with both optimal binding properties and cellular functionality. Such techniques enable easy and rapid identification of artificial nucleic acid aptamers, and would contribute to the advancement of aptamer research in the future.

Our hybrid-type SELEX could be particularly useful in the discovery of artificial nucleic acid aptamers. CE-SELEX filters multiple artificial nucleic acid libraries and explores aptamers based on their binding properties, whereas cell-SELEX screens aptamers based on their functionality in cells. This process maximizes CE capabilities. CE-SELEX is a promising choice for the pre-screening of various modified libraries. With CE, it is possible to comprehensively screen artificial nucleic acid libraries with a wider range of modification patterns in terms of functional group diversity than in the case of conventional methods. Thus, this technology can be used to determine the appropriate artificial nucleic acid libraries suitable for the target protein at the initial selection stage. This should further improve the success probability of SELEX. Despite a growing number of relevant studies [53], the process of identifying promising modification patterns of nucleic acids is not straightforward. Various ideas for modified libraries of artificial nucleic acids have been suggested, but they relied on multiple SELEX procedures or design-testing cycles for a post-modification approach, as the identification of the optimal approach for the target is still a laborious task. Because of its exceptionally high detection sensitivity and partitioning efficiency, CE can detect only modest target-aptamer interactions; this allowed Berezovski et al. to develop a 1-step process for aptamer selection [54]. As the peak area of the target-aptamer complex correlates with the abundance ratio of the active species in the complex library, the complex peak ratio might be a good indicator of the success rate of each modified library. This phenomenon was observed in our previous report, where a higher complex peak was obtained with the initial library, containing more high-affinity aptamers than other libraries [30]. We introduced U^trp^ or U^trp^/C^aa^ into the initial aptamer libraries, to increase the probability of TROP2 binding and to augment capacity for cellular internalization. The U^trp^-modified initial library alone produced a detectable target-aptamer complex peak (Figure 1B). To neutralize the negative charges of aptamers, which may prevent them from crossing the cell membrane, C^aa^ was introduced, as it has a unit positive charge. However, the target-aptamer complex peak could not be detected through the CE analysis under our experimental conditions for the U^trp^/C^aa^ double-modified library (Figure 1B). The combination of U^trp^ and C^aa^ might not result in an ideal modification pattern for TROP2 binding, which highlights the importance of accessing the modification patterns of initial libraries prior to the full-scale SELEX procedure in order to increase the success rate, reduce the cost, and shorten the time of the experiment.

We were able to generate anti-TROP2 aptamers with double-digit nanomolar-level affinities after nine rounds of CE-SELEX; however, the aptamers selected via CE-SELEX alone showed poor internalization by MCF-7 cells. There have been reports of anti-TROP2 aptamers with greater binding affinities than our aptamer; however, to the best of our knowledge, the cell internalization of those aptamers had not been evaluated [38]. On the other hand, the aptamer selected using hybrid-type SELEX showed higher degree of cell internalization; nevertheless, its TROP2 binding affinity was less than that observed using CE-SELEX alone. Thus, it is crucial to thoroughly examine whether aptamer capacity for cell internalization activity is TROP2-dependent. To verify this point, knockdown experiments using siRNA or binding experiments using cells with different expression levels of TROP2 protein could be considered [35,55]. For further verification, improving internalization by cells is desirable, because in this study, the degree of cell internalization of the anti-TROP2 aptamer was only ~2-fold higher than that of the control. To address this issue, we can use elegant techniques, including truncation, maturation, or multimerization, to generate highly active aptamers [56,57]. With the clinical success of antibody-drug conjugates, drug delivery technologies utilizing various molecules (peptides, aptamers, antibody fragments, etc.) targeting TROP2 may emerge in the near future. The findings of this study provide important insights into future drug delivery using TROP2 protein-targeting aptamers.

CE is a powerful pre-screening tool with minimal human bias, because CE analysis does not require pre-immobilization of the target protein and can be easily performed in a semi-automatic manner. One potential limitation of our method could be that it depends on the availability of membrane proteins. Usually, recombinant membrane proteins are relatively harder to obtain in terms of solubility and stability compared to soluble proteins. Various techniques have been developed for addressing this issue, such as nanodisc technology [58,59]. If we could obtain a variety of membrane proteins by fully utilizing these techniques, it would accelerate our CE-based screening technology. Therefore, a much larger number of aptamers could be obtained more easily by automating the selection process compared to the number afforded by conventional experiments that rely on manual labor.

Our hybrid SELEX could be a powerful platform for obtaining cell-internalizing aptamers for intracellular delivery of therapeutic oligonucleotides in a rational and mechanism-based manner, because aptamers obtained with this platform have guaranteed binding affinity to the selected targets and high capacity for internalization. Owing to the similar chemistry of aptamers and current therapeutic oligonucleotides, such as ASOs or siRNA, it is easy to design and synthesize various aptamer-oligonucleotide conjugates. In our previous report, we described the intracellular delivery of ASOs against human lung cancer cell line A549 using U^trp^-modified aptamers obtained with cell-SELEX [43]. We revealed that aptamer-ASO conjugates enhanced ASO internalization by cells without impairing their RNA-degradating activity. One of the challenges of the delivery of ASOs by aptamers is the endosomal/lysosomal escape of ASOs [48,60]. Therefore, we would like to further improve our platform, so that we can obtain aptamers that promote endosomal escape after internalization by cells. Such aptamers will enable cell-specific intracellular delivery of oligonucleotides and could be used for next-generation therapeutic oligonucleotides.

## 5. Conclusions

We successfully identified artificial nucleic acid aptamers that could bind to TROP2 and be internalized by MCF-7 cells. All reported aptamers were discovered through the hybrid selection approach, and they contained a tryptamine modification in their deoxyuridine nucleotide (U^trp^). Our results suggest that a combination of the nucleobase modification and the hybrid-type SELEX protocol could provide a useful platform for the discovery of aptamers for other cell surface targets with desired cellular functions. We conclude that the addition of the functional screening step in the screening process is helpful for the discovery of functional aptamers. Studies evaluating our TROP2-binding aptamer as a targeting tool or a drug carrier for cancer treatment are underway.

## Figures and Tables

**Table 1:** Sequence and binding affinities of aptamers discovered using CE-SELEX.

ID	Random Region Sequence (N30, 5′ to 3′)	Copies in Round 9	*K*_d_ (nM)		**T (U^trp^)Count**
			TROP2 FcHis	FcHis	
Tac-A1	**T**C**TT**ACCG**TTT**CC**T**CG**T**GCC**TT**G**TTTT**CGC	0.82%	103 ± 11	804 ± 98.8	14
Tac-A2	**T**CG**TT**CC**T**G**TT**G**T**G**TT**CCC**TT**C**T**CC**T**C**T**G**T**	0.62%	74 ± 8.4	>1000	15
Tac-A3	**T**CG**TTT**AC**T**G**TT**CCCC**T**CC**T**CC**TT**CCC**TTT**	0.43%	100 ± 7.9	>1000	14
Tac-A4	**TT**G**TT**CCCCC**TTT**GCC**TTT**C**TTT**CCCC**T**C**T**	0.40%	72 ± 5.4	>1000	15
Tac-A5	**T**C**T**G**TT**CCG**T**G**TT**CG**TT**CC**TTT**CC**TT**G**TT**G	0.37%	50 ± 6.9	>1000	16

Affinity of binding to TROP2 FcHis or FcHis was measured using CE. *K*_d_, mean ± SD; *n* = 3; T = U^trp.^

**Table 2:** Sequence information obtained with hybrid-type SELEX.

ID	Random Region Sequence (N30, 5′ to 3′)	Copies in Round 9 (in Rd 9b)	*K*_d_ (nM)		**T (U^trp^)Count**
			TROP2 FcHis	FcHis	
Tac-B1	**T**GC**T**G**TT**G**T**CACC**T**GCC**T**CG**T**C**T**CCC**T**CG**T**	0.16% (0.35%)	153 ± 13	>1000	11
Tac-B2	**TT**CCC**T**CC**T**C**T**G**TT**G**TT**CCCCCC**T**CC**T**C**T**C	0.03% (0.32%)	89 ± 6.0	>1000	12
Tac-B3	**T**GGGG**T**GG**T**GG**T**G**T**GGG**T**GGGGG**TTT**G**TT**C	0.25% (0.17%)	187 ± 8.4	>1000	11

Affinity of binding to TROP2 FcHis or FcHis was measured with CE. *K*_d_, mean ± SD; *n* = 3; Rd = Round; T = U^trp.^

## Supplementary Materials

The following supplementary materials are available online at https://www.mdpi.com/article/10.3390/pharmaceutics13060888/s1: Figure S1: Capillary electrograms of the binding assays, Figure S2: Evaluating the binding affinity of random libraries and ssDNA-neg to TROP2 FcHis or FcHis proteins, Figure S3: Electrophoretic mobility shift assay for evaluating the binding of TROP2 aptamer, ssDNA-neg, and random libraries to the TROP2 FcHis or FcHis, Figure S4: Expression of TROP2 protein on the MCF-7 cell surface, Figure S5: Comparison of sequences in the final rounds of CE-SELEX and cell-SELEX using NGS data, Table S1: Names and sequences of oligonucleotides used in this study, Table S2. Conditions of cell-SELEX experiment with MCF-7 cells.
