# Supplementary material for: Hybrid-Type SELEX for the Selection of Artificial Nucleic Acid Aptamers Exhibiting Cell Internalization Activity
Source: Pharmaceutics. 2021 Jun 15;13(6):888. doi: 10.3390/pharmaceutics13060888 (PMC8232673; doi:10.3390/pharmaceutics13060888)
Supplement: Supplementary file 1 [file pharmaceutics-13-00888-s001.zip › pharmaceutics-1194899 - supplementary.pdf]

pharmaceutics

MDPI

Supplementary Materials: Hybrid-Type SELEX for the Selection of Artificial Nucleic Acid Aptamers Exhibiting Cell Internalization Activity

Hiro Uemachi, Yuuya Kasahara, Keisuke Tanaka, Takumi Okuda, Yoshihiro Yoneda and Satoshi Obika

Supplementary Figures

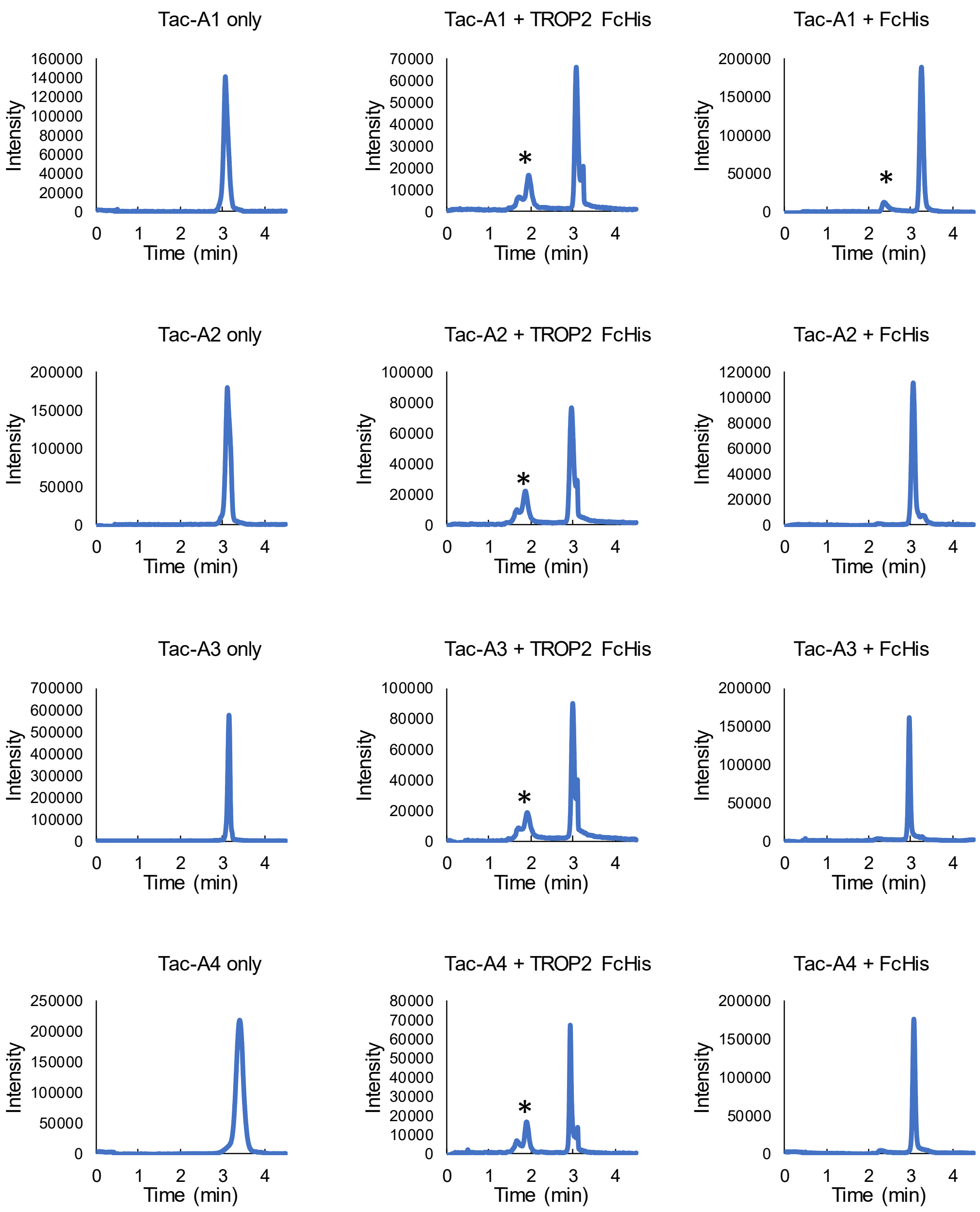

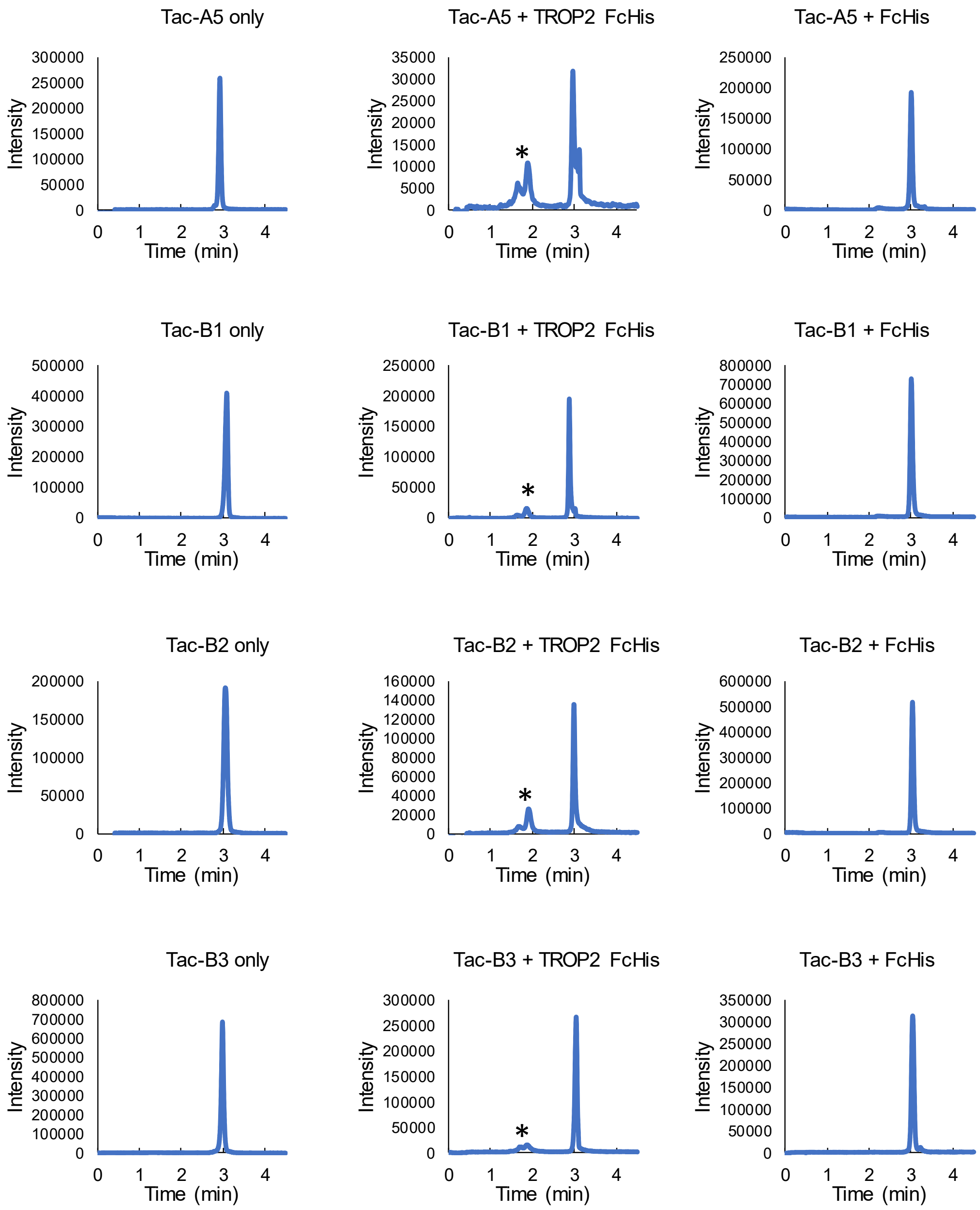

Figure S1. Capillary electrograms of the binding assays. TacA1~A5 and TacB1~B3 (10 nM) were incubated with or without 4-fold equivalents of TROP2 FcHis or FcHis protein, respectively. All electrograms recorded fluorescent intensity of FAM group at 5′ end of each aptamer. The asterisk indicates the peak of the protein–aptamer complex.

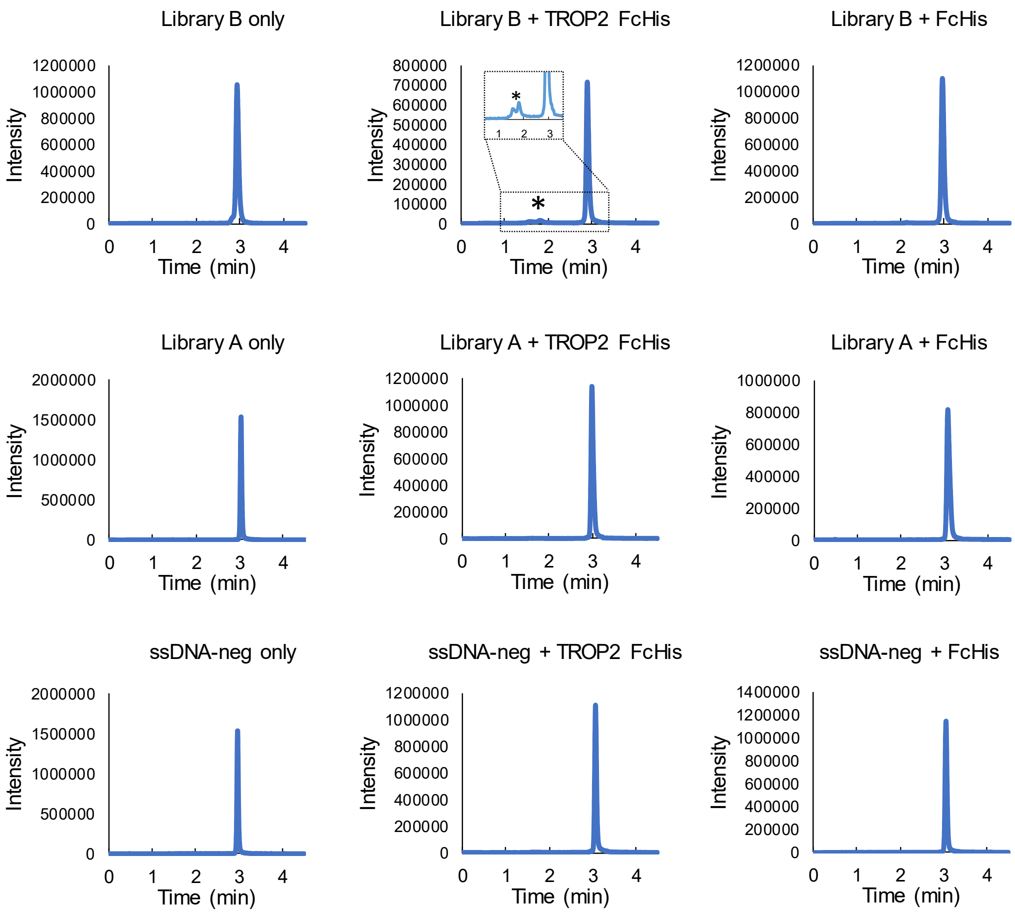

Figure S2. Evaluating the binding affinity of random libraries and ssDNA-neg to TROP2 FcHis or FcHis proteins. The CE-based binding assay was carried under the same conditions as that in the Figure S1. Random libraries or ssDNA-neg (10 nM) was incubated with or without 4-fold equivalents of TROP2 FcHis or FcHis protein, respectively. The binding affinity was evaluated by detecting the fluorescence from each DNA sample. The asterisk indicates the peak of the protein–aptamer complex.

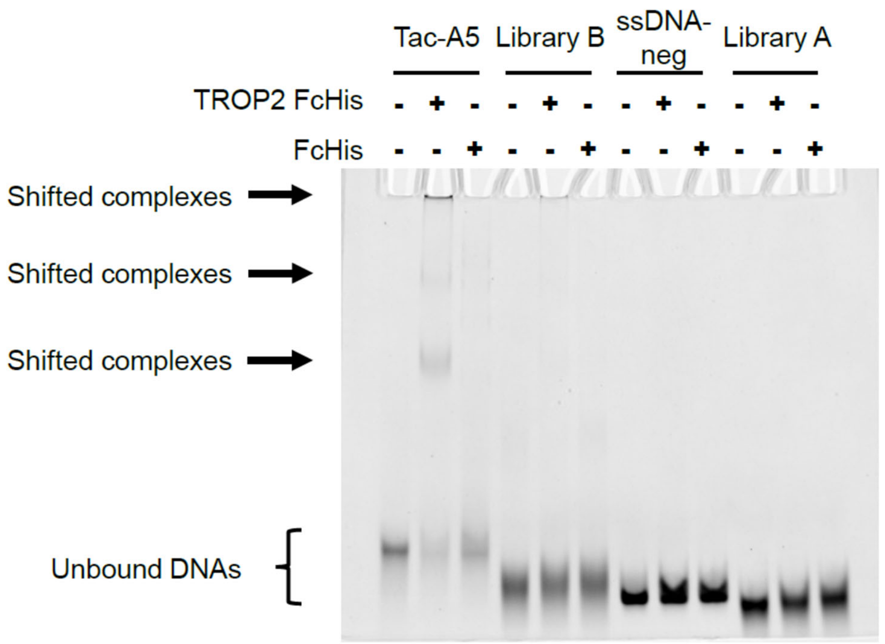

Figure S3. Electrophoretic mobility shift assay for evaluating the binding of TROP2 aptamer, ssDNA-neg, and random libraries to the TROP2 FcHis or FcHis. The aptamer, ssDNA-neg, and random libraries (20 nM) were incubated respectively with TROP2 or FcHis proteins (100 nM) in the binding buffer (20 mM Tris-HCl buffer [pH 7.4], 10 mM NaCl, 1 mM $MgCl_2$, 2 mg/mL salmon sperm DNA, and 0.05% Tween 20) at 37 °C for 30 min. The mixture of DNA and protein was loaded on a 5% native gel with TB (Tris-borate, 0.5×) as the running buffer. The samples were run under native conditions at 200 V and 25 °C for 38 min. Fluorescence of each DNA sample was detected. The data are representative of three independent experiments.

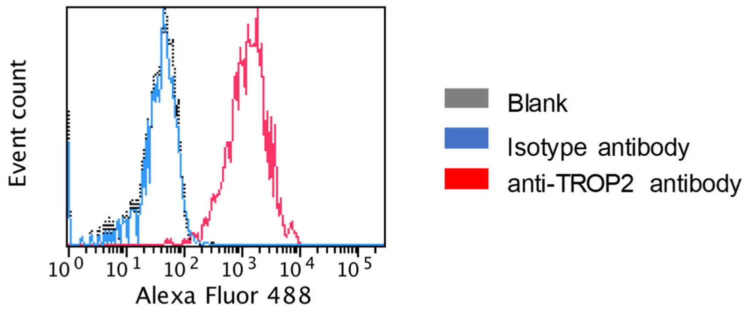

Figure S4. Expression of TROP2 protein on the MCF-7 cell surface. Cells were washed with PBS and detached by treatment of 0.05% trypsin (Nacalai Tesque, Japan) The resulting cells were suspended with culture and adjusted to a concentration of 1×10^6 cells/mL. Cells were incubated with primary antibodies (mouse anti-TROP2 (R&D systems, #MAB650) 1:500 or mouse isotype control (R&D systems, #MAB003) 1:500) at 4 °C for 30 min followed by incuvation with secondary antibody (Goat anti-Mouse IgG, Alexa Fluor 488 (Invitrogen, #A-11029) 1:500). The samples were then measured using a LSRFortessa X-20™ (BD Biosciences, San Jose, CA, USA) and the mean fluorescence intensity (at least 10,000 events) was analyzed using a FCSalyzer Version 0.9.16-alpha.

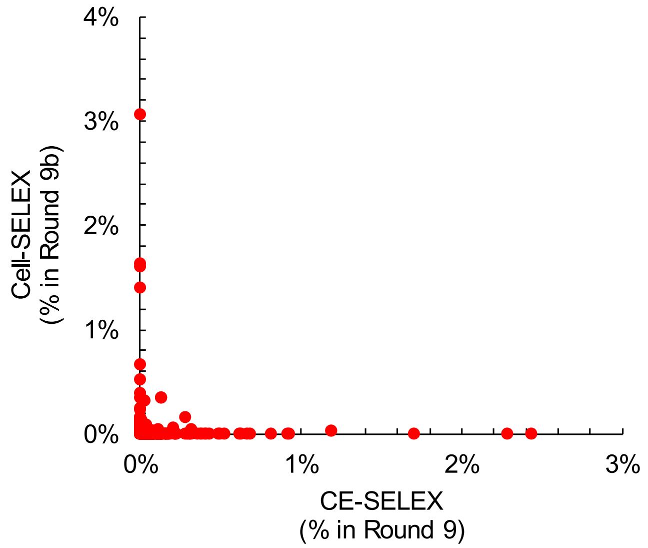

Figure S5. Comparison of sequences in the final rounds of CE-SELEX and cell-SELEX using NGS data. We plotted the percentage of each cluster in the final round 9 of CE-SELEX and final round 9b of cell-SELEX.

Supplementary Tables

Table S1. Names and sequences of oligonucleotides used in this study.

Name	Sequence (5′ to 3′)
P1F	FAM-TCGCCTTGCCGGATCGCAGA
P1P	P-TCGCCTTGCCGGATCGCAGA
P2H_C3	AAAAAAAAAAAAAAAXGGTGTCAGGCTCACGGACCA
T2H	HEX-GGTGTCAGGCTCACGGACCANNNNNNNNNNNNNNNNNNNNNNNNNNNNNNTCTGCGATCCGGCAAGGCGA
Library A (DNA library)	FAM-TCGCCTTGCCGGATCGCAGANNNNNNNNNNNNNNNNNNNNNNNNNNNNNNTGGTCCGTGAGCCTGACACC
Library B	FAM-TCGCCTTGCCGGATCGCAGANNNNNNNNNNNNNNNNNNNNNNNNNNNNNNU^{trp}GGU^{trp}CCGU^{trp}GAGCCU^{trp}GACACC
Library C	FAM-TCGCCTTGCCGGATCGCAGANNNNNNNNNNNNNNNNNNNNNNNNNNNNNNU^{trp}GGU^{trp}C^{aa}C^{aa}GU^{trp}GAGC^{aa}C^{aa}U^{trp}GAC^{aa}AC^{aa}C^{aa}
Forward primer	TCGCCTTGCCGGATCGCAGA
Reverse primer	GGTGTCAGGCTCACGGACCA
Constant Region 1	TCGCCTTGCCGGATCGCAGA
Constant region 2	TGGTCCGTGAGCCTGACACC
ssDNA_neg	FAM-TCGCCTTGCCGGATCGCAGACTTCCCCCCACTCCACTTCTTACTCCTTTCTGGTCCGTGAGCCTGACACC

FAM: Fluorescein amidite, P: phosphate, X: C3 linker, HEX: Hexachloro-fluorescein

Table S2. Conditions of cell-SELEX experiments with MCF-7 cells.

	**Rd6b**	**Rd7b**	**Rd8b**	**Rd9b**
LL97A cell count	$1.2×10^6$	$1.2×10^6$	$1.2×10^6$	$1.2×10^6$
Incubation time (min)	60	60	30	30
Aptamer concentration (nM)	50	50	50	50
Wash times	3	4	5	6
